# Pulmonary MALT Lymphoma has variable features on CT

**DOI:** 10.1038/s41598-019-45144-9

**Published:** 2019-06-17

**Authors:** Wen Deng, Ying Wan, Jian-qun Yu

**Affiliations:** 1 Department of Radiology, West China Hospital, Sichuan University, 37# Guo Xue Xiang, Chengdu, Sichuan 610041 China; 2 Department of Pathology, West China Hospital, Sichuan University, 37# Guo Xue Xiang, Chengdu, Sichuan 610041 China

**Keywords:** Cancer imaging, Cancer imaging, B-cell lymphoma, B-cell lymphoma

## Abstract

Pulmonary mucosa-associated lymphoid tissue (MALT) lymphoma is the most common primary pulmonary lymphoma. There are limited studies on imaging features of pulmonary MALT lymphoma. We present the computed tomography (CT) manifestations of pulmonary MALT lymphoma and the correlation between CT manifestations and clinical characteristics. Patients (n = 53) with histologically confirmed pulmonary MALT lymphoma who underwent chest CT scanning were retrospectively analyzed. Evaluated findings included distribution of pulmonary lesions, morphological pattern of appearance, contrast enhancement features, size, presence of thoracic lymphadenopathy, and secondary associated features. Pulmonary MALT lymphoma was observed in multiple (79%) and bilateral (66%) disease with random distribution (≥70%) of pulmonary lesions. The most frequent morphological pattern was consolidation (n = 33, 62%), followed by nodule (n = 23, 43%) and mass (n = 11, 21%). Common associated features were air bronchograms and bronchiectasis, especially cystic bronchiectasis and angiogram sign. Asymptomatic patients had less consolidation and bronchiectasis than did symptomatic patients. Cystic bronchiectasis was only observed in the symptomatic group. In conclusion, pulmonary MALT lymphoma manifests as diverse patterns on CT scans. Consolidation combined with cystic bronchiectasis was a characteristic late sign, which may assist in differential diagnosis. High-resolution CT images and multiplanar reconstruction techniques are helpful for accurately determining imaging manifestations.

## Introduction

Pulmonary mucosa-associated lymphoid tissue (MALT) lymphoma is rare but is the most common primary pulmonary lymphoma^1–3^. MALT lymphoma is classified as extra nodal marginal zone, B-cell lymphoma^3–5^. It arises from bronchus-associated lymphoid tissue (BALT), which is a component of the pulmonary lymphoid system. BALT is located beneath areas of specialized bronchial epithelium comprising non-ciliated flattened epithelial cells, known as M cells, throughout the airways^6–9^. BALT is absent in the normal healthy adult and appears with antigenic stimulation, including inflammation, smoke, collagen vascular disease, infection, and AIDS^6,7,10,11^.

Patients with pulmonary MALT lymphoma are often asymptomatic; the disease is typically detected incidentally during imaging^10,12–14^. Treatment is usually with chemotherapy or immunotherapy. The prognosis is good, with 5-and 10-year survival rates >80%^13,15–17^. Therefore, the differentiation of pulmonary MALT lymphoma from other lung diseases is important for patient management.

In recent years, computed tomography (CT) technology and image post-processing technology have developed rapidly. With the shortening of scanning time, improvement of anatomical resolution of CT images, and application of multiplanar reconstruction techniques, CT examination has become a powerful tool in the evaluation of pulmonary lesions. The imaging manifestations of pulmonary MALT lymphoma on CT are diversiform. The most common findings are consolidation with air bronchograms, pulmonary mass, mass-like areas of consolidation, and multiple pulmonary nodules^14,17–23^. To date, the distribution and CT manifestations of pulmonary MALT lymphoma have not been fully elucidated. The purpose of this study was to describe the distribution and CT manifestations of pulmonary MALT lymphoma lesions in histologically diagnosed cases.

## Methods

### Patients

Our institutional review board approved our retrospective study with a waiver of informed consent. We reviewed all pathological records in our hospital from January 2009 to December 2017and identified 86 patients with pathologic diagnoses of pulmonary MALT lymphoma. The histopathological diagnosis was based on the World Health Organization criteria. One patient who had a history of follicular lymphoma for 8 years was excluded. Chest imaging obtained before pathological diagnosis was available in 53 of 85 patients. Among the 53 patients enrolled in the study, specimens obtained for histological analysis were as follows: CT-guided percutaneous lung biopsy (n = 18), bronchoscopic biopsy (n = 18), and surgical resection (n = 17). If there were two or more reexaminations before pathological diagnosis in a patient, only the first examination was included in this study.

### CT technique

Due to the retrospective nature of the study, scanning protocols varied. CT scanning was performed on one of six machines ranging from 16-detector to 128-detector CT scanners (Philips Medical Systems, Best, the Netherlands [n = 15] or Siemens Medical Systems, Erlangen, Germany[n = 38]). Scanning was performed from the level of the superior margin of the thoracic cavity to the level of the inferior margin of the thoracic cavity. Forty of the 53 patients underwent contrast-enhanced examination. For contrast-enhanced examination, patients received 80–100 mL of nonionic contrast medium (iopamidol,350 mg/mL; Bracco Sine Pharmaceutical Corp. Ltd, Shanghai, China) + 30 mL physiological saline infused using a power injector (Stellant, Medrad, Inianola, USA) at a flow rate of 2.5–3.5 mL/s. Imaging started 25 s after completion of intravenous contrast medium injection. In all patients, CT scans were obtained with 5-mm-thick sections at 5 mm intervals. Scanning parameters were 100–120 kVp and 70–200 mA. Sections of 1 mm thickness were reconstructed in 35 of 53 patients by applying a sharp reconstruction algorithm and lung window setting (window width,1500 HU; window level, −700 HU).

### Imaging analysis

All chest CT imaging was retrospectively and independently reviewed at a workstation (Leonardo, Siemens Medical Solutions) by two experienced chest radiologists with more than 5 years of experience. Multiplanar reconstruction techniques were used in all 1mm-thick section series of images to determine the imaging manifestations. Where there was disagreement on CT findings, a consensus opinion was obtained.

Lesion distribution and laterality (unilateral or bilateral) in the lungs were analyzed on thoracic CT images. For all patients, the presence of lesions in each lung lobe was recorded. The predominant distribution of pulmonary lesions was classified on the longitudinal plane into upper (i.e., higher than hilum), lower (i.e., lower than hilum), or random; and on the transverse plane into central (i.e., central one-third), peripheral (i.e., peripheral one-third), or random. The distribution of nodules was classified as subpleural, peribronchovascular, subpleural plus peribronchovascular, or random. Solitary and non-solitary pulmonary lesions were recorded. Multiform pulmonary lesions were defined as three or more primary pulmonary lesions present simultaneously.

CT imaging was analyzed for major pulmonary lesions as the presence of areas of consolidation, mass, nodule, and ground-glass opacity (GGO). Areas of consolidation were defined as homogeneous increase in pulmonary parenchymal attenuation that obscures the margins of vessels and airway walls^24^. Mass was defined as an opacity greater than 3 cm in diameter^24^. Nodule was defined as a rounded or irregular opacity, well or poorly defined, measuring up to 3 cm in diameter^24^. GGO was defined as an area of hazy increased lung opacity with preservation of bronchial and vascular margins, and was considered to be present only when it was visible on high-resolution 1 mm CT sections^24^. For areas of consolidation, the presence of secondary associated features as air bronchogram, bronchiectasis, and cystic bronchiectasis was described. Cystic bronchiectasis was defined as localized abnormal dilation of bronchi that manifest as round or spherical bronchi on cross-sectional image. The presence of a positive angiogram sign was described in patients who underwent contrast-enhanced examinations. The CT angiogram sign was defined as the demonstration of enhancing vascular structures within areas of consolidation^19^. For mass and nodule lesions, the presence of associated features such as lobulation, spiculation, the halo sign, and the pleural indentation sign was described. Other CT manifestations, including lymphadenopathy, pleural thickening or effusion, reticular pattern, and pulmonary cyst were also recorded. Lymphadenopathy was defined as lymph nodes in hilar greater than 3 mm or in mediastinum greater than 1 cm in short-axis diameter^24^. Pulmonary cyst was defined as a round parenchymal low-attenuating area with a well-defined interface with normal lung, occurring without associated pulmonary emphysema^24^.

For patients with the presence of areas of consolidation, mass, or nodules who also underwent contrast enhanced CT, pre-enhancement attenuation, enhanced attenuation, and the degree of enhancement were measured in Hounsfield units. Only the largest lesion was measured in each patient. Two separate measurements of mean attenuation using circular region of interest (ROI) were made for each lesion, and the average of the values was recorded. The area of the lesion ROI was more than 60% of that of the entire lesion for mass and solid nodules. For areas of consolidation, the diameter of the lesion ROI was no less than 1 cm. Care was taken to avoid vasculature and airway structures within the lesion. ROI circles in unenhanced images were copied and pasted from the same site in contrast-enhanced images.

### Statistical analysis

All statistical analyses were performed on a personal computer using Statistical Package for Social Science (SPSS) version 22.0 for Windows (IBM, Chicago, IL). The numeric variables are described as mean, standard deviation, minimum, and maximum. The descriptive analysis of categorical variables comprised the calculation of simple and relative frequencies. The correlation between CT manifestations and clinical characteristics was analyzed using Fisher’s exact test. Statistical significance was denoted as *p* value less than 0.05.

## Results

### Patient’s features

We evaluated 53 patients with histologically proven pulmonary MALT lymphoma (27males and 26 females). Mean age was 56.1 ± 12.9 years (range 25–86 years). Of the 53 patients, 36 (67.9%) were symptomatic, including 28 patients who had a history of respiratory symptoms such as dyspnea or cough, 10 patients who had weight loss, and six patients who complained of chest pain. Seventeen patients (32.1%) were asymptomatic.In 16 of 17 asymptomatic patients, lung abnormalities were detected by annual physical examination and in one patient by radiograph due to a fall. The mean duration from the onset of symptoms or identification of an asymptomatic pulmonary lesion to the histological diagnosis being made was 167.14 ± 432.34 days. Six of the 53 patients (11%) had diagnosis duration greater than 3 months due to various factors including: non-diagnostic biopsy specimens, patients declining biopsy or surgery, and slow progression of pulmonary lesions such that the possibility of malignant disease was not considered in the first place.

The associated conditions were as follows: none of these patients had AIDS, two patients had rheumatoid arthritis, one patient had Sjögren syndrome, and one patient was simultaneously diagnosed with gastrointestinal MALT lymphoma during hospitalization. Fourteen patients were current cigarette smokers for 5–50 years (mean = 27.2 years) that smoked 2–40 cigarettes/day (mean = 16.5 cigarettes); five were previous smokers who had stopped 2 months-15 years previously (mean = 5 years), 29 were non-smokers, and smoking history was unavailable in the remaining five patients. Patients’ clinical characteristics at diagnosis are summarized in Table 1.

**Table 1:** Clinical Characteristics of 53 patients at diagnosis.

Characteristics	N. (%)
Age mean (range)	56.1 (25–86)
Male/Female	27/26
**Symptoms**	
Respiratory symptoms	28 (52.8%)
Weight loss	10 (18.9%)
Chest pain	6 (11.3%)
Asymptomatic	17 (32.1%)
**Associated conditions**	
rheumatoid arthritis	2
Sjögren syndrome	1
**Smoking history**	
Current/previous smoker	19 (35.8%)
Non-smoker	29 (54.7%)

### Distribution and CT manifestations

The distribution of pulmonary lesions in the 53 patients is shown in Table 2. There were 11 cases (21%) identified as solitary lesions. The other 42 cases (79%) were identified as non-solitary lesions. Pulmonary lesions were bilateral in 35 cases (66%), accounting for more than half of the 53 cases. In the longitudinal distribution, most cases were randomly distributed (30%). Similarly, in the transverse distribution, most cases were randomly distributed (74%). There was no predominant central distribution in any of the cases. The majority of peripheral distribution cases were solitary lesions (n = 9); only five cases of non-solitary lesions had peripheral distribution. For pulmonary lobe involvement, there were 14 cases of single lobe involvement. Among the 49 multiple lobe involvement cases, 23 patients had involvement of all lung lobes. For distribution of nodules, there were 14 cases with a multiple nodule pattern, of which four were randomly distributed, eight had peribronchovascular distribution, and two had subpleural plus peribronchovascular distribution. None of the 14 multiple nodule cases were purely of subpleural distribution.

**Table 2:** Lesion Distribution of Pulmonary MALT lymphoma.

Distribution	N. (%)
**Laterality**	
Unilateral	18 (34%)
Bilateral	35 (66%)
**Transverse**	
Upper	6 (11%)
Lower	10 (19%)
Random	37 (30%)
**Longitudinal**	
Central	0
Peripheral	14 (26%)
Random	39 (74%)

All types of major pulmonary lesions were observed in 53 patients. The major pulmonary lesions manifested as consolidation, nodules, mass, or GGO. These CT features existed in various patterns classified as single pattern, double pattern, and multiform lesions on CT images. The major patterns and second associated features are shown in Tables 3 and 4.

**Table 3:** Major Patterns of Pulmonary MALT Lymphoma.

Single pattern	N. (%)	Double pattern	N. (%)	Multiform lesions	N. (%)
Con.	23 (44%)	Con. + Nodule	7 (13%)	Con. + Mass + Nodule	2 (4%)
Nodule	9 (17%)	Con. + Mass	1 (2%)		
Mass	5 (10%)	Nodule + Mass	2 (4%)		
GGO	1 (2%)	Nodule + GGO	2 (4%)		

Note: Con. = consolidation; GGO = ground glass opacity.

**Table 4:** Second Associated Features of Pulmonary MALT Lymphoma.

Major Pattern	N. (%)	Associated Feature	N. (%)
Consolidation	33 (62%)	air bronchogram	33 (100%)
		bronchiectasis	21 (64%)
		cystic bronchiectasis	11 (33%)
Nodule	23 (43%)	lobulation	2 (8%)
		spiculation	1 (4%)
		halo sign	6 (26%)
Mass	11 (21%)	lobulation	3 (27%)
		spiculation	2 (18%)
		PIs.	1 (9%)

Note: PIs. = pleural indentation sign.

**Figure 1:**
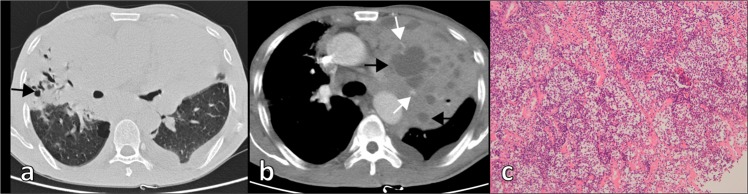
CT imaging showed consolidation and associated features in pulmonary MALT lymphoma in a 66-year-old man. **(a)** Contrast-enhanced CT imaging showed consolidation with air bronchogram in the right middle lobe. There was cystic bronchiectasis (black arrows). There was a pulmonary cyst in the right lower lobe. **(b)** Contrast-enhanced CT imaging showed consolidation in the left upper lobe. There were lots of fluid-filled cysts (black arrows) of dilated airway in the area of consolidation. The vasculature structures adjacent to the cystic bronchiectasis were intact (white arrows). Lymphadenopathy in mediastinum and pleural effusion were also found in this patient. **(c)** Light microscopic image of CT-guided percutaneous lung biopsy showed diffuse infiltration of lymphoid cells. (Hematoxylin-Eosin stain; 200 × magnification).

**Figure 2:**
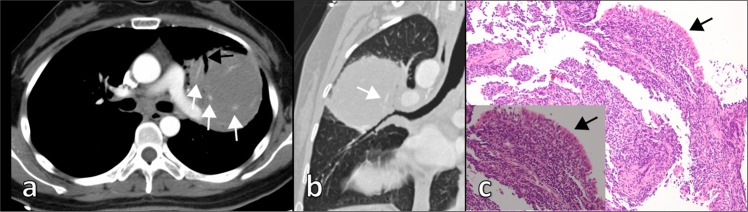
Pulmonary MALT lymphoma in a 27-year-old woman. **(a)** Thoracic axial CT imaging showed a mass in the left upper lobe with air bronchograms (black arrow). Positive angiogram sign (white arrows) were also found in the mass with the pulmonary vessels compressed and displaced. **(b)** Curved plane reconstruction image revealed air bronchogram in the mass. There was irregular bronchial wall thickening and positive angiogram sign (white arrow). **(c)** Light microscopic image of bronchoscopic biopsy showed diffuse infiltration of lymphoid cells within mucosa of bronchus (black arrow). (Hematoxylin-Eosinstain, 200 × magnification; 400 × magnification for inset).

**Figure 3:**
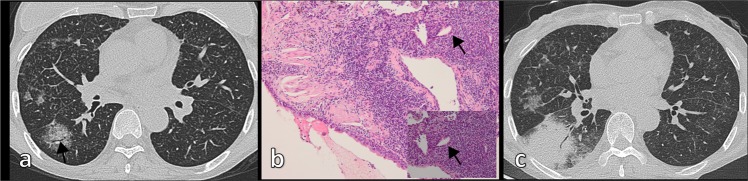
Pulmonary MALT lymphoma in a 54-year-old woman. **(a)** Axial HRCT imaging showed multiple ground-glass opacities (GGO) with air bronchogram (black arrow). The bronchus was lack of tapering. There were also micronodules which showed a peribronchovascular distribution. **(b)** Light microscopic image of lung biopsy demonstrated lymphocytic infiltration into the bronchus mucosa and peribronchus intersitium. Also note the small lymphoepithelial cells in the bronchiolar mucosa (black arrow). (Hematoxylin-Eosin stain, 200 × magnification; 400 × magnification for inset) **(c)** The patient received chemotherapy (rituximab). However, one year later, thoracic CT imaging showed areas of consolidation with air bronchogram in the right lower lobe where there was GGO previously, which means the condition progressed.

Consolidation (Fig. 1) was the most frequent CT feature and existing pattern of pulmonary lesions (n = 33, 62%), followed by nodule (n = 23, 43%) and mass (n = 11, 21%) (Fig. 2). GGO was the least common pattern (n = 3, 6%) (Fig. 3). Most cases were single pattern (n = 38, 72%). All 33 patients with consolidation had air bronchogram, and 23 patients had mild bronchiectasis. We also observed cystic bronchiectasis in 11 patients; all of whom were respiratory symptomatic patients. In these patients, the cyst of the dilated bronchus may contain air, fluid, or both; with air-fluid level. The size of the cyst of the dilated bronchus ranged from 6 mm to 45 mm in longitudinal diameter (mean = 20.3 ± 13.5 mm). Double pattern was detected in 12 patients. Nodules in seven patients and mass in one patient were detected in coexistence with consolidation. The angiogram sign was observed in 24 of 30 patients (80%) who received intravenous contrast.

The sign of nodules and mass were detected in 23 patients and 11 patients, respectively. The size of nodule and mass ranged from 3 mm to 77 mm in longitudinal diameter. For nodule pattern, the most frequently associated feature was the halo sign (n = 6, 26%). Lobulation and spiculation were rare signs in nodule and mass. Reticular pattern was identified in four of 53 patients (8%), including one case combined with honeycombing sign. Multiple pulmonary cysts were detected in three of 53 patients (6%). Thoracic lymphadenopathy was identified in 19 of 53 patients (36%). Pleural effusion was observed in 17 of 53 patients (32%), including four cases combined with pleural thickening.

Unenhanced attenuation, enhanced attenuation, and enhancement were successfully measured in 38 patients (Table 5). The mean enhancement of all measured lesions was 37.52 ± 26.84 HU, ranging from 5.47 HU to 123.33 HU. Nodule and mass lesions were significantly enhanced. Consolidation lesions were also significantly enhanced, but the degree of enhancement was slightly lower than that of the nodule and mass lesions.

**Table 5:** CT Numbers (in Hounsfield Units) for pulmonary MALT lymphoma.

CT Pattern (N.)	Unenhanced attenuation	Enhanced attenuation	Enhancement*
Consolidation (23)	45.96 ± 14.26	77.57 ± 28.15	31.61 ± 21.29
Nodule and mass (15)	−8.33 ± 74.36	39.40 ± 44.75	47.73 ± 33.37

Note: N. = number of patients; Numbers are mean ± standard deviation.

*Values represent maximum change in attenuation value between unenhanced and enhanced images.

### Correlation between CT manifestations and clinical characteristics

Patients were divided into asymptomatic and symptomatic groups based on the absence or presence of symptoms, respectively. The correlation between CT manifestations and clinical characteristics is summarized in Table 6. There were no differences in nodule pattern, mass pattern, or GGO pattern between the two groups with the exception of consolidation and bronchiectasis. Asymptomatic patients had less consolidation and bronchiectasis than did symptomatic patients. Cystic bronchiectasis was only observed in the symptomatic group. There were no significant differences in CT manifestations between smokers and non-smokers.

**Table 6:** Correlation between CT Manifestations and Clinical Characteristics.

CT Pattern	Asym.	Sym.	*p* value	Smoker	Non-smoker	*p* value
Con.	7	26	0.031*	14	15	0.111
Nodule	9	14	0.252	8	15	0.361
Mass	5	6	0.237	4	5	0.512
GGO	1	2	0.695	0	3	0.211
Bro.	4	19	0.042*	11	10	0.097
Cystic Bro.	0	11	0.008*	6	4	0.132

Note: Asym. = asymptomatic group; Sym. = symptomatic group; GGO = ground glass opacity; Con. = consolidation; Bro. = bronchiectasis.

*There was a significant difference (*p* < 0.05) between asymptomatic patients and symptomatic patients. Smoker group including current smokers and previous smokers.

## Discussion

Pulmonary MALT lymphoma, although a rare disease, is the most common primary pulmonary lymphoma, accounting for up to 90% of primary pulmonary lymphomas. Patients tend to present around the 6th decade, however, there are also reports of young patients^1^. Due to the lack of specific biological marker for the diagnosis, being asymptomatic or lacking characteristic imaging features, pulmonary MALT lymphoma is often misdiagnosed or has delayed diagnosis. In this study, we analyzed the clinical and CT features of 53 patients with pulmonary MALT lymphoma as well as the correlation between clinical and imaging features. The results demonstrated that 68% of patients with pulmonary MALT lymphoma had various symptoms, whereas 32% of patients lacked symptoms. Multiple and bilateral distribution of lesions on CT images was the major characteristic of this disease. In both longitudinal and transverse distributions, most cases were randomly distributed (≥70%), consistent with previous studies^20,21^.

According to past studies^17–22^ describing imaging manifestations of pulmonary MALT lymphoma, the patterns of pulmonary lesions observed on CT scans varied, including consolidation with air bronchograms, nodules, mass, GGO, and diffuse interstitial lung disease patterns. In our study, the most common major patterns were consolidation, followed by nodule and mass. GGO (3/53) and reticular pattern (4/53)were relatively rare in our study, which is similar to the findings of most other studies. However, Bae *et al*.^20^ reported diffuse interstitial lung disease patterns as a major pattern of pulmonary MALT lymphoma. In our study, reticular pattern was rare. Further, reticular pattern was never observed in isolation and was always accompanied with consolidation, mass, or multiple nodules. Thus, we did not classify this type of pattern as a major pattern of pulmonary MALT lymphoma.

In our study, consolidation (n = 33, 62%) was the most frequent CT feature and co-existing pattern of pulmonary lesions. All 33 patients with consolidation had air bronchogram, and 23 had mild bronchiectasis. Furthermore,11 patients had cystic bronchiectasis; all of them had respiratory symptoms. These results agree with previous findings^17,19,22,23^. For patients with areas of consolidation pattern, it was unclear whether the degree of enhancement of the lesions contributed to differential diagnosis. As pulmonary inflammation with high and low biological activities receive various contributions from the pulmonary circulation depending on inflammatory activity, there was a varied degree of enhancement of consolidation resulting from inflammation^25,26^. It is challenging to differentiate consolidation of pulmonary MALT lymphoma from inflammation by means of degree of enhancement. The cyst of the dilated bronchus may contain air, fluid, or both. Lee *et al*.^22^ reported bubble-like radiolucencies present in 50% of patients in their study of 10 pathologically proven BALT lymphoma. Although they used “bubble-like radiolucency” to describe this imaging phenomenon, it was actually cystic bronchiectasis. King *et al*.^19^ reported three cases of cavitation which were later histologically proven to be cystic bronchiectasis in a study of 24 patients. Thus, over the past 10 years of relevant studies, cystic bronchiectasis may be mistaken for cavitation due to the limitations of CT technology and lack of multiplanar reconstruction techniques. According to past research on CT findings and pathological correlations of pulmonary MALT lymphoma^17^ there was no destruction of the bronchial wall or tumor necrosis in the dilated bronchus and/or bronchioles. Dilated airways may result from alveolar collapse or destruction of parenchyma adjacent to airways due to lymphomatous infiltration.

Owing to previous studies of dynamic CT, malignant pulmonary nodules usually have a higher degree of enhancement on contrast enhanced CT than do benign pulmonary nodules. As such, 15 or 20 HU was set as the cutoff value for differentiation. Most malignant nodules had peak enhancement approximately 2 minutes after the administration of contrast medium^25,27–29^. In our study, the enhancement in most lesions ranged from 30 HU to 50 HU with an average of 37.52 HU. For patients with mass or nodule pattern, the degree of enhancement of the lesions was helpful to differentiate them from benign lesions. The degree of enhancement of pulmonary MALT lymphoma was similar to malignant pulmonary nodules based on previous studies.

In our study, all cases with cystic bronchiectasis were respiratory symptomatic patients, a phenomenon that has not been reported previously. Pulmonary MALT lymphoma is often asymptomatic when pulmonary lesions are observed on CT for the first time. Further, there were significant differences in cystic bronchiectasis between asymptomatic and symptomatic groups in our study. Therefore, we speculate that cystic bronchiectasis is a late imaging manifestation of pulmonary MALT lymphoma. The speculated chronological changes of consolidation in pulmonary MALT lymphoma were as follows: consolidation with air bronchogram was an early imaging manifestation; then, bronchiectasis occurred (most of them were cylindrical and varicose bronchiectasis); finally, cystic bronchiectasis increasingly appeared. Although there were no significant differences in CT manifestations between smokers and non-smokers, the proportion of consolidation, bronchiectasis, and cystic bronchiectasis in smokers was higher than that in non-smokers, indicating that smoking maybe a factor accelerating the progress of disease course.

The differential diagnosis for pulmonary MALT lymphoma includes entities that can manifest consolidation, nodule, mass, or GGO containing areas of low attenuation at CT. These include infection, bronchioloalveolar carcinoma, granulomatosis with polyangiitis (formerly Wegener granulomatosis), sarcoidosis, and perilymphatic spread of metastatic disease. Chronic consolidations containing an air bronchogram should be considered diagnosis of organizing pneumonia, bronchioloalveolar carcinoma, or pulmonary MALT lymphoma^17,30^. Symptoms of fever and leukocytosis suggest infection. If anti-infective therapy is ineffectual, pulmonary MALT lymphoma should be considered as a possible diagnosis. The possibility of metastatic disease should be considered when patients have a history of prior malignancy. Along with granulomatosis, polyangiitis, bronchioloalveolar carcinoma, and abscesses, pulmonary MALT lymphoma should also be included in the differential diagnosis of a nodule or mass combined with an area of low attenuation^17,22,31,32^. True cavitation may be observed in granulomatosis, polyangiitis, or bronchioloalveolar carcinoma, mimicking cystic bronchiectasis which is often detected in pulmonary MALT lymphoma. Furthermore, the continuity of the inner wall of the cyst of dilated airways and the inner wall of the airway without adjacent structure destruction maybe observed on high-resolution CT imaging using multiplanar reconstruction techniques. This differs from the cavity resulting from necrosis due to airway or vasculature destruction. CT angiogram sign may also be helpful in differentiating pulmonary MALT lymphoma from bronchioloalveolar carcinoma. The edge of the vasculature structures was smooth and clear in CT angiogram sign in pulmonary MALT lymphoma, while bronchioloalveolar carcinoma had an irregular and hazy vessel border. Nodular lymphoid hyperplasia and lymphocytic interstitial pneumonia should also be included in the differential radiologic diagnosis of pulmonary MALT lymphoma. However, these types of pulmonary lymphoid disorders are difficult to histologically differentiate^33,34^. Furthermore, pulmonary MALT lymphoma can coexist with lymphocytic interstitial pneumonia^1,13,35–37^. Thus, an indolent nature, bilaterality, multiplicity, and diverse patterns of parenchymal abnormality favor a diagnosis of pulmonary MALT lymphoma^20^. The final diagnosis requires either transbronchial or surgical biopsy.

In conclusion, pulmonary MALT lymphoma can manifest as diverse patterns of pulmonary parenchymal abnormality at CT, but multiple bilateral lesions are common. Areas of consolidation or nodule are the main patterns. Bronchiectasis (especially cystic bronchiectasis) and CT angiogram sign are characteristic associated features which may be helpful in differential diagnosis. High-resolution CT images and multiplanar reconstruction techniques are helpful for accurately determining imaging manifestations.

## Data Availability

The datasets generated during and/or analyzed during the current study are available from the corresponding author on reasonable request.
